# PmtA functions as a ferrous iron and cobalt efflux pump in *Streptococcus suis*

**DOI:** 10.1080/22221751.2019.1660233

**Published:** 2019-08-30

**Authors:** Chengkun Zheng, Mengdie Jia, Miaomiao Gao, Tianyu Lu, Lingzhi Li, Pingping Zhou

**Affiliations:** a Joint International Research Laboratory of Agriculture and Agri-Product Safety, The Ministry of Education of China, Yangzhou University, Yangzhou, People’s Republic of China; b Jiangsu Key Laboratory of Zoonosis, Yangzhou University, Yangzhou, People’s Republic of China; c State Key Laboratory of Agricultural Microbiology, Huazhong Agricultural University, Wuhan, People’s Republic of China

**Keywords:** Metal toxicity, metal efflux pump, oxidative stress resistance, virulence, *Streptococcus suis*

## Abstract

Transition metals are nutrients essential for life. However, an excess of metals can be toxic to cells, and host-imposed metal toxicity is an important mechanism for controlling bacterial infection. Accordingly, bacteria have evolved metal efflux systems to maintain metal homeostasis. Here, we established that PmtA functions as a ferrous iron [Fe(II)] and cobalt [Co(II)] efflux pump in *Streptococcus suis*, an emerging zoonotic pathogen responsible for severe infections in both humans and pigs. *pmtA* expression is induced by Fe(II), Co(II), and nickel [Ni(II)], whereas PmtA protects *S. suis* against Fe(II) and ferric iron [Fe(III)]-induced bactericidal effect, as well as Co(II) and zinc [Zn(II)]-induced bacteriostatic effect. In the presence of elevated concentrations of Fe(II) and Co(II), Δ*pmtA* accumulates high levels of intracellular iron and cobalt, respectively. Δ*pmtA* is also more sensitive to streptonigrin, a Fe(II)-activated antibiotic. Furthermore, growth defects of Δ*pmtA* under Fe(II) or Co(II) excess conditions can be alleviated by manganese [Mn(II)] supplementation. Finally, PmtA plays a role in tolerance to H_2_O_2_-induced oxidative stress, yet is not involved in the virulence of *S. suis* in mice. Together, these data demonstrate that *S. suis* PmtA acts as a Fe(II) and Co(II) efflux pump, and contributes to oxidative stress resistance.

## Introduction

Transition metals such as iron, manganese, zinc, and copper are nutrients essential for life, and a variety of enzymes require a metal cofactor for their catalytic activity [1,2]. Decreasing the availability of these metals plays an important role in the strategy used by the host immune system to limit bacterial growth [3]. As a countermeasure, bacteria employ various mechanisms to acquire metals from diverse sources [1]. While acquiring metals is crucial for bacterial survival, an excessive accumulation of these metals can be toxic to cells [4]. Emerging evidence indicates that imposed metal toxicity is another host defense strategy against bacterial pathogens [1]. For example, neutrophils can mobilize zinc in response to *Streptococcus pyogenes* infection [5]. Consequently, bacteria have evolved complex systems to maintain metal homeostasis, such as efflux or sequestration of metals [6]. Moreover, there is mounting evidence that metal homeostasis, maintained via metal efflux pumps, plays a critical role in bacterial physiology and pathogenesis [5–11].

*Streptococcus suis* is an important swine pathogen, causing meningitis, septicemia, pneumonia, endocarditis, and arthritis [12]. It is also an emerging zoonotic agent, which has been associated with meningitis, septicemia, and other infections in humans [12–14]. Currently, 29 serotypes have been proposed for *S. suis* [15]. Among them, *S. suis* serotype 2 (*S. suis* 2) is the most frequently isolated worldwide and the most commonly involved in disease in both pigs and humans [12]. By 2013, *S. suis* had resulted in more than 1600 human cases of infection worldwide, with the majority reported in Asia [14]. Of note, a total of 240 human cases with 53 deaths were recorded in China during two large outbreaks of *S. suis* 2 infection, which occurred in 1998 and 2005 [16,17]. In recent years, sporadic human cases of *S. suis* infection have been frequently reported worldwide [18–22], indicating that *S. suis* is a persistent threat to public health.

To date, the metal efflux systems of *S. suis* have received little attention. Indeed, a single cation efflux family protein, MntE, has been identified as a manganese [Mn(II)] export system and is involved in *S. suis* virulence [23]. Recently, a PerR-regulated P_1B-4_-type ATPase (PmtA) has been reported to act as a ferrous iron [Fe(II)] efflux pump and to contribute to oxidative stress resistance and virulence in *S. pyogenes* [10,11]. Moreover, PfeT, the PmtA homolog of *Bacillus subtilis*, is known to protect bacteria from iron intoxication [7], while FrvA, the PmtA homolog of *Listeria monocytogenes*, is critical for its virulence [8]. These findings indicate that in certain bacteria, Fe(II) efflux and homeostasis are important for bacterial survival and pathogenesis. In *S. suis*, iron sequestration by Dpr plays a central role in intracellular iron homeostasis [24,25]. However, whether an Fe(II) efflux mechanism exists in *S. suis* remains unclear.

In this study, we focused on the PmtA homolog of *S. suis* and confirmed its role in Fe(II) and cobalt [Co(II)] efflux. We also investigated the role of *S. suis* PmtA in oxidative stress resistance and virulence. Our findings revealed that PmtA plays a role in oxidative stress resistance, yet has no effect on *S. suis* virulence.

## Materials and methods

### Bacterial strains, plasmids, primers, and culture conditions

Bacterial strains and plasmids used in this study are listed in Table S1. Primers are listed in Table S2. *S. suis* 2 strain SC19 [26] and its isogenic derivatives were routinely cultured at 37°C in Tryptic Soy Broth (TSB) or on Tryptic Soy Agar (TSA; Becton, Dickinson and Company) supplemented with 10% (vol/vol) newborn bovine serum. *Escherichia coli* strain DH5α was grown in Luria–Bertani (LB) broth or on LB agar. When required, spectinomycin was added at 50 and 100 μg/ml for *E. coli* and *S. suis*, respectively.

### Quantitative gene expression analysis

The SC19 strain was grown to mid-exponential phase (OD600 =0.6) and divided into eight aliquots, seven of which were treated for 15 min with 1 mM FeSO_4_, 1 mM Fe(NO_3_)_3_, 0.25 mM CoSO_4_, 1 mM MnSO_4_, 1 mM NiSO_4_, 0.5 mM CuSO_4_, or 0.1 mM ZnSO_4_, respectively. The remaining aliquot was supplemented with deionized water (H_2_O) and served as the control. For each sample, total RNA was extracted using the Eastep Super total RNA isolation kit (Promega). RNA (500 ng) was converted to cDNA using the PrimeScript RT reagent Kit (TaKaRa). The MIQE guidelines [27] were followed for quantitative PCR (qPCR) analysis. qPCR was performed on a StepOnePlus Real-Time PCR System (Applied Biosystems) using TB Green Premix Ex Taq II (TaKaRa). Melting curve analysis was performed to detect the specificity of the products. The reaction efficiency was evaluated by using serially diluted cDNA as the template. The relative gene expression level was assessed using the 2^-ΔΔCT^ method [28] with 16S rRNA as the reference gene.

### Construction of a pmtA deletion mutant and functional complementation

Allelic exchange using a pSET4s suicide vector [29] was performed to generate a *pmtA* deletion mutant (Δ*pmtA*) in the SC19 background. The complementation strain was constructed using the pSET2 vector, as described previously [30].

### Growth curve analyses

- Metal toxicity assay. The wild-type (WT), Δ*pmtA*, and CΔ*pmtA* strains grown to exponential phase were diluted in fresh medium supplemented with various concentrations of individual metals. The cells were grown in flat-bottom 96-well plates (200 μl/well) at 37°C, and the optical density at 595 nm (OD_595_) was measured hourly using a CMax Plus plate reader (Molecular Devices). The metal tested here included Fe(II) (FeSO_4_), ferric iron [Fe(III)] [Fe(NO_3_)_3_], Co(II) (CoSO_4_), Mn(II) (MnSO_4_), nickel [Ni(II)] (NiSO_4_), copper [Cu(II)] (CuSO_4_), and zinc [Zn(II)] (ZnSO_4_). Since Fe(II) rapidly oxidizes to Fe(III), a fresh FeSO_4_ solution was prepared before each use, and 1 g/l of trisodium citrate dihydrate (TCD) was added to the medium containing FeSO_4_ to reduce iron precipitation [7].
- Oxidative stress resistance assay. Overnight cultures of the WT, Δ*pmtA*, and CΔ*pmtA* strains were grown to exponential phase in the presence of 2 mM FeSO_4_. The cells were harvested and resuspended in an equal volume of fresh medium. The suspension was then diluted in fresh medium supplemented with oxidative agents (H_2_O_2_, diamide, or paraquat). The cells were grown in 96-well plates at 37°C, and the OD_595_ was measured hourly. In a second experiment, the WT, Δ*pmtA*, and CΔ*pmtA* strains grown to exponential phase were diluted in fresh medium supplemented with oxidative agents and Co(II). The cells were grown in 96-well plates at 37°C, and the OD_595_ was measured hourly.
- Rescue of the Δ*pmtA* mutant by Mn(II) supplementation. The WT, Δ*pmtA*, and CΔ*pmtA* strains grown to exponential phase were diluted in fresh medium supplemented with 4 mM FeSO_4_ or 0.25 mM CoSO_4_, and increasing concentrations of MnSO_4_. The cells were grown in 96-well plates at 37°C, and the OD_595_ was measured hourly.
- Streptonigrin sensitivity assay. The WT, Δ*pmtA*, and CΔ*pmtA* strains grown to exponential phase were diluted in fresh medium supplemented with 2 mM FeSO_4_ and increasing concentrations of streptonigrin. The cells were grown in 96-well plates at 37°C, and the OD_595_ was measured hourly.

### Spot dilution assays

The WT, Δ*pmtA*, and CΔ*pmtA* strains were grown to exponential phase and diluted in fresh medium supplemented with 4 mM Fe(NO_3_)_3_, 0.125 mM ZnSO_4_, or H_2_O. The cultures were further incubated at 37°C. At 3 and 6 h, aliquots were serially diluted 10-fold up to 10^−5^ dilution, and 5 μl of each dilution was spotted onto ager plates. The plates were photographically documented after approximately 18 h of incubation at 37°C. This assay was also performed for H_2_O_2_ sensitivity. The WT, Δ*pmtA*, and CΔ*pmtA* strains were grown to exponential phase in the presence of 2 mM FeSO_4_. The cells were harvested and resuspended in an equal volume of fresh medium. The suspension was diluted in fresh medium supplemented with either 0.5 mM H_2_O_2_ or H_2_O, and then incubated at 37°C. At 4 and 6 h, aliquots were serially diluted 10-fold up to 10^−5^ dilution, and 5 μl of each dilution was spotted onto ager plates. The plates were photographically documented following incubation.

In another assay, the WT, Δ*pmtA*, and CΔ*pmtA* strains were grown to mid-exponential phase. Each culture was then divided into six equal parts, which were treated with 4 mM FeSO_4_, 1 g/l TCD, 0.25 mM CoSO_4_, 4 mM Fe(NO_3_)_3_, 0.125 mM ZnSO_4_, or H_2_O, respectively. At 2 and 3 h, aliquots were serially diluted 10-fold up to 10^−5^ dilution, and 5 μl of each dilution was spotted onto ager plates. The plates were then photographically documented following incubation.

### Intracellular metal content analysis by ICP-OES

The WT, Δ*pmtA*, and CΔ*pmtA* strains were grown to early exponential phase (OD_600 _= 0.3). Each culture was then divided into four aliquots, which were treated for 2 h with 2 mM FeSO_4_, 1 g/l TCD, 0.125 mM CoSO_4_, or H_2_O, respectively. The cells were harvested, washed three times with PBS supplemented with 250 mM EDTA, and three times with PBS. A sample was taken, and the total protein concentration was determined using a Bradford Protein Assay Kit (Sangon Biotech). The remaining samples were centrifuged, resuspended in 66% nitric acid, digested at 70°C for 48 h, diluted to 2% nitric acid with H_2_O, and analyzed by inductively coupled plasma-optical emission spectroscopy (ICP-OES). The metal content was expressed as μg of metal per g of protein.

### Mouse infections

Animal studies were approved by the Animal Welfare and Ethics Committees of Yangzhou University. Forty female BALB/c mice (4–6 weeks old) were randomly divided into four groups. The mice in three of the groups were intraperitoneally infected with 300 μl of PBS containing 3×10^8^ CFU of the WT, Δ*pmtA*, and CΔ*pmtA* strains, respectively. The mice in the remaining group were intraperitoneally mock-infected with 300 μl of PBS and served as a control. The mice were monitored twice daily over seven days for clinical symptoms and survival rates.

### Protein sequence analysis and statistical analysis

Protein sequence alignments were performed using Clustal Omega (https://www.ebi.ac.uk/Tools/msa/clustalo/) and processed using ESPript 3.0 (http://espript.ibcp.fr/ESPript/ESPript/). The structure of PmtA was predicted using SWISS-MODEL (https://www.swissmodel.expasy.org/). The promoter of *pmtA* was predicted using BPROM (http://linux1.softberry.com/berry.phtml).

Statistical analyses were performed using GraphPad Prism 5. The differences in gene expression were analyzed by 1-way analysis of variance with Bonferroni’s post-test, while the differences in intracellular metal concentrations were analyzed by the two-tailed paired *t*-test. The mice survival was analyzed using the Log-rank test.

## Results

### S. suis PmtA is a homolog of Fe(II) efflux pumps found in several gram-positive bacteria

In the *S. suis* SC19 genome, the product of the B9H01_RS01605 locus is annotated as a heavy metal translocating P-type ATPase [26]. This protein exhibits 60%, 48%, and 46% amino acid sequence identity with Fe(II) efflux pumps from *S. pyogenes* (PmtA) [10,11], *L. monocytogenes* (FrvA) [8], and *B. subtilis* (PfeT) [7], respectively. Hence, this protein was designated as PmtA, and we hypothesized that *S. suis* PmtA was implicated in Fe(II) efflux. Multiple sequence alignment revealed that PmtA possesses two metal-binding motifs, a Ser-Pro-Cys (SPC) motif and an His-Glu-Gly-Ser-Thr (HEGST) motif located in transmembrane region 4 and 6, respectively (Figure S1). These two motifs are characteristic of the P_1B4_-type ATPases [31,32]. Located 162 bp upstream of *pmtA* is the gene encoding the PerR regulator [33], which was also referred to as Zur [34] or Fur [35]; while 134 bp downstream of *pmtA* is a gene encoding a hypothetical protein (Figure S2). Furthermore, BLASTN analyses revealed that *pmtA* is present in all complete *S. suis* genomes, with 91% to 100% sequence identity at the nucleotide level (Table S3).

### PmtA expression is upregulated in response to Fe(II), Co(II), and Ni(II)

**Figure 1.**
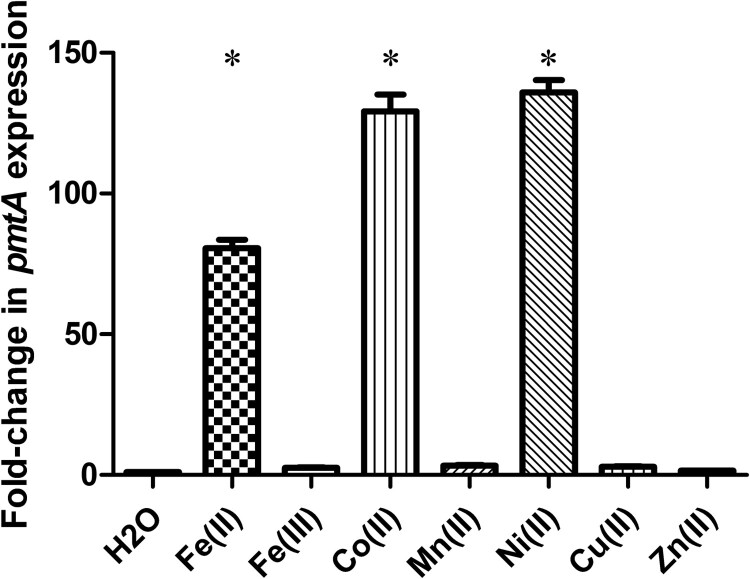
*pmtA* expression in *S. suis* grown in the presence of various metals. Graph data are mean values ± standard deviation (SD) from three biological replicates. * indicates *P* < 0.05.

To assess the involvement of PmtA in metal efflux, we first examined *pmtA* relative gene expression in response to various metals, including Fe(II), Fe(III), Co(II), Mn(II), Ni(II), Cu(II), and Zn(II). Compared to H_2_O treatment, the transcript levels of *pmtA* were 80-fold higher when the SC19 strain was treated with 1 mM Fe(II) (Figure 1). Furthermore, the expression level of *pmtA* increased approximately 130- and 136-fold after treatment with Co(II) and Ni(II), respectively (Figure 1). In contrast, no significant difference in *pmtA* expression was observed when the SC19 strain was treated with Fe(III), Mn(II), Cu(II), and Zn(II) (Figure 1). Altogether, these results suggest that *S. suis pmtA* expression is upregulated in response to an excess of Fe(II), Co(II), or Ni(II), which led us to speculate that PmtA may be involved in the efflux of Fe(II), Co(II), and Ni(II).

### PmtA is involved in S. suis growth under iron, cobalt or zinc excess conditions

To explore the role of PmtA in *S. suis* physiology, we constructed an in-frame deletion mutant of *pmtA* (Δ*pmtA*) and the corresponding complementation strain (CΔ*pmtA*). The two strains were confirmed by PCR (Figure S3A), reverse transcription PCR (RT–PCR) (Figure S3B), and DNA sequencing (data not shown). Under normal growth conditions, *pmtA* expression in CΔ*pmtA* was approximately 160-fold higher than in the WT strain (Figure S3C).

**Figure 2.**
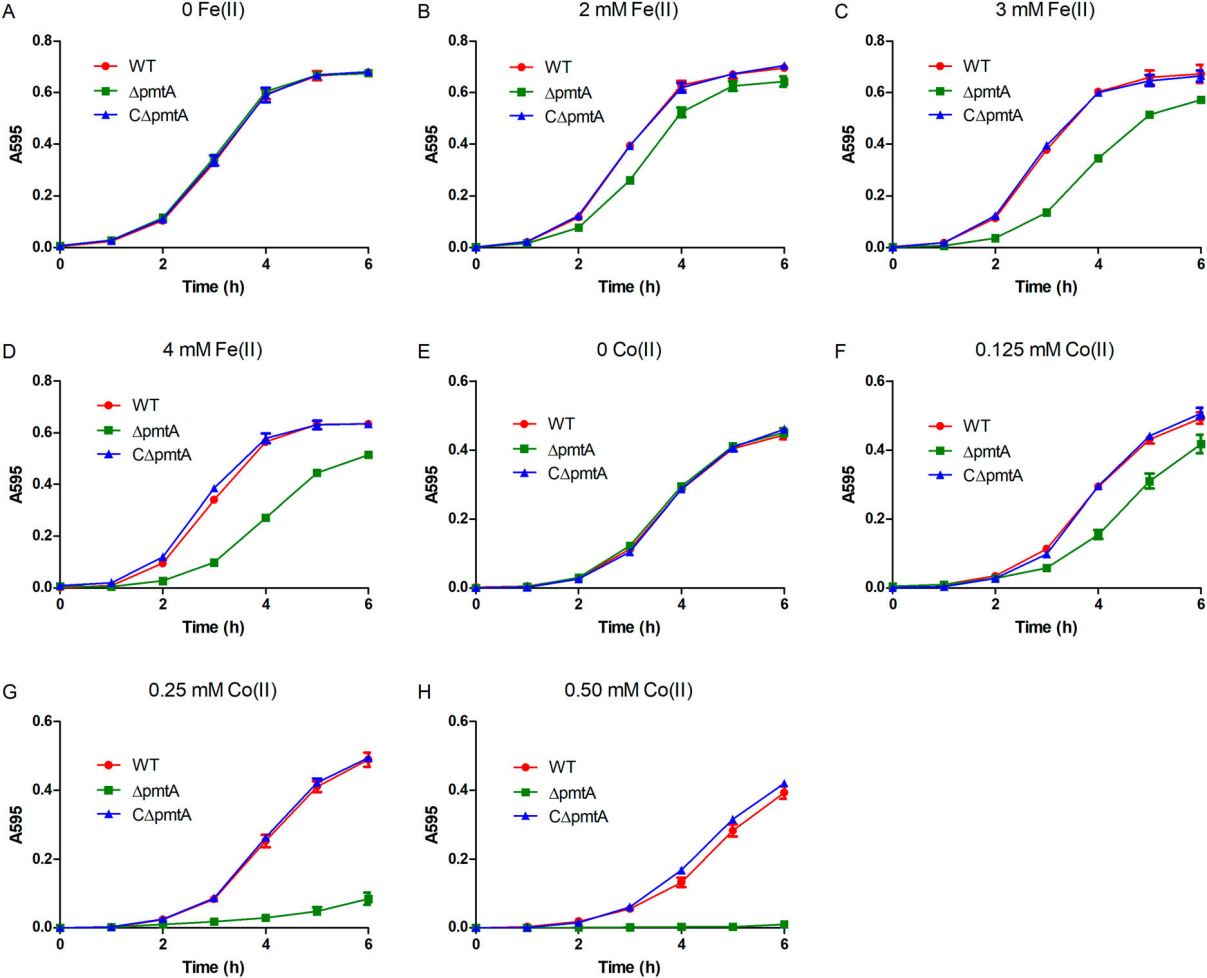
PmtA is involved in *S. suis* resistance to iron or cobalt excess. (A–D) The WT, Δ*pmtA*, and CΔ*pmtA* strains were grown in the absence (A) and presence of 2 mM (B), 3 mM (C), or 4 mM Fe(II) (D). (E-H) Growth curves in the absence (E) and presence of 0.125 mM (F), 0.25 mM (G), or 0.50 mM Co(II) (H). Graphs data are mean values ± SD from three wells.

In the absence of Fe(II), all three strains exhibited similar growth rates (Figure 2A). However, when exposed to various concentrations of Fe(II), Δ*pmtA* exhibited growth inhibition, and the level of inhibition correlated with the increase in Fe(II) concentration (Figure 2B–D). The growth defect of Δ*pmtA* was even more prominent with the increase of Co(II) (Figure 2E–H), while slightly impaired growth was observed when Δ*pmtA* was exposed to Fe(III) or Zn(II) (Figure S4A–C). In line with the growth curve results, spot dilution assays showed that Δ*pmtA* formed less colonies than did the WT strain when grown in the presence of Fe(III) or Zn(II) (Figure S5). The expression of PmtA in CΔ*pmtA* rescued the growth defect of Δ*pmtA* under iron, cobalt, or zinc excess conditions (Figure 2, S4A–C, and S5), confirming that this phenotype is due to the deletion of *pmtA*. Growth curve analyses performed in the presence of Ni(II), Mn(II), and Cu(II) showed no major difference between the WT, Δ*pmtA*, and CΔ*pmtA* strains (Figure S4D–F).

**Figure 3.**
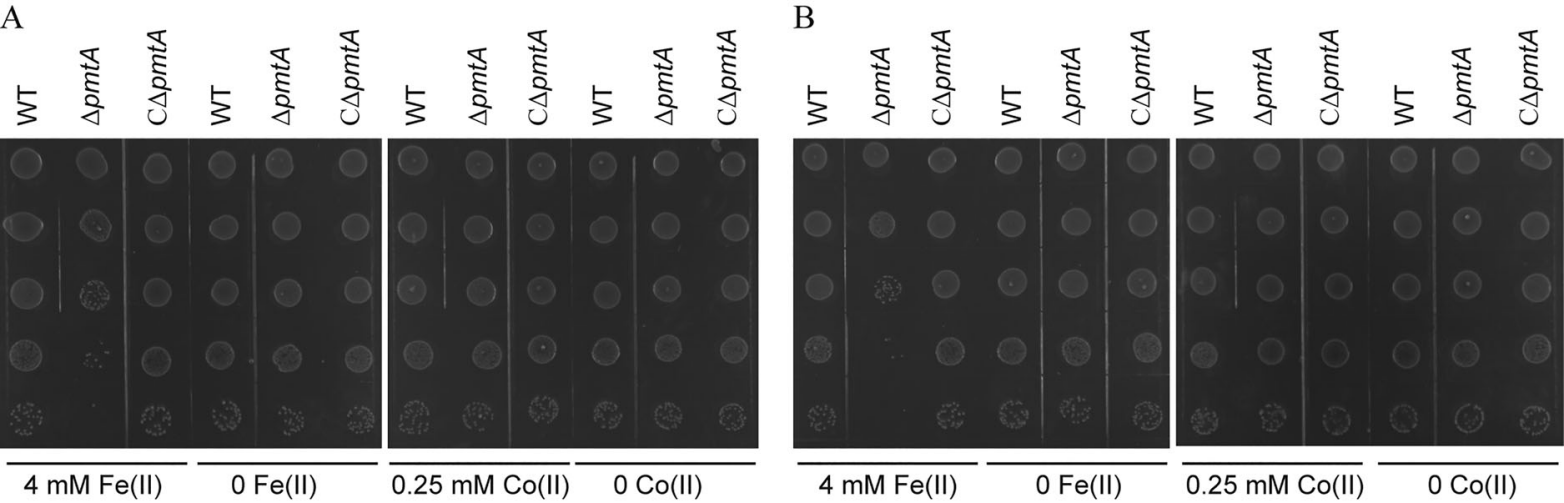
Spot dilution assays of the *S. suis* strains treated with Fe(II) or Co(II). The WT, Δ*pmtA*, and CΔ*pmtA* strains were grown to mid-exponential phase. Each culture was next treated with 4 mM Fe(II), 1 g/l TCD, 0.25 mM Co(II), or H_2_O, respectively. At 2 h (A) and 3 h (B), aliquots were serially diluted 10-fold up to 10^–5^ dilution, and 5 μl of each dilution was spotted onto ager plates. The graphs are representative of three independent experiments.

To further asses the role of PmtA in resistance to Fe(II), Co(II), Fe(III), and Zn(II), spot dilution assays were performed. Following treatment for 2 h with Fe(II) (Figure 3A) or Fe(III) (Figure S6A), Δ*pmtA* formed less colonies than did the WT and CΔ*pmtA* strains. The effect was even more prominent following 3 h of treatment (Figures 3B and S6B). However, the three strains displayed similar abilities to form colonies after treatment with Co(II) (Figure 3) or Zn(II) (Figure S6).

Collectively, our results indicate that PmtA protects *S. suis* against Fe(II) and Fe(III)-induced bactericidal effect, as well as Co(II) and Zn(II)-induced bacteriostatic effect.

### PmtA deletion leads to increased levels of intracellular iron and cobalt

**Figure 4.**
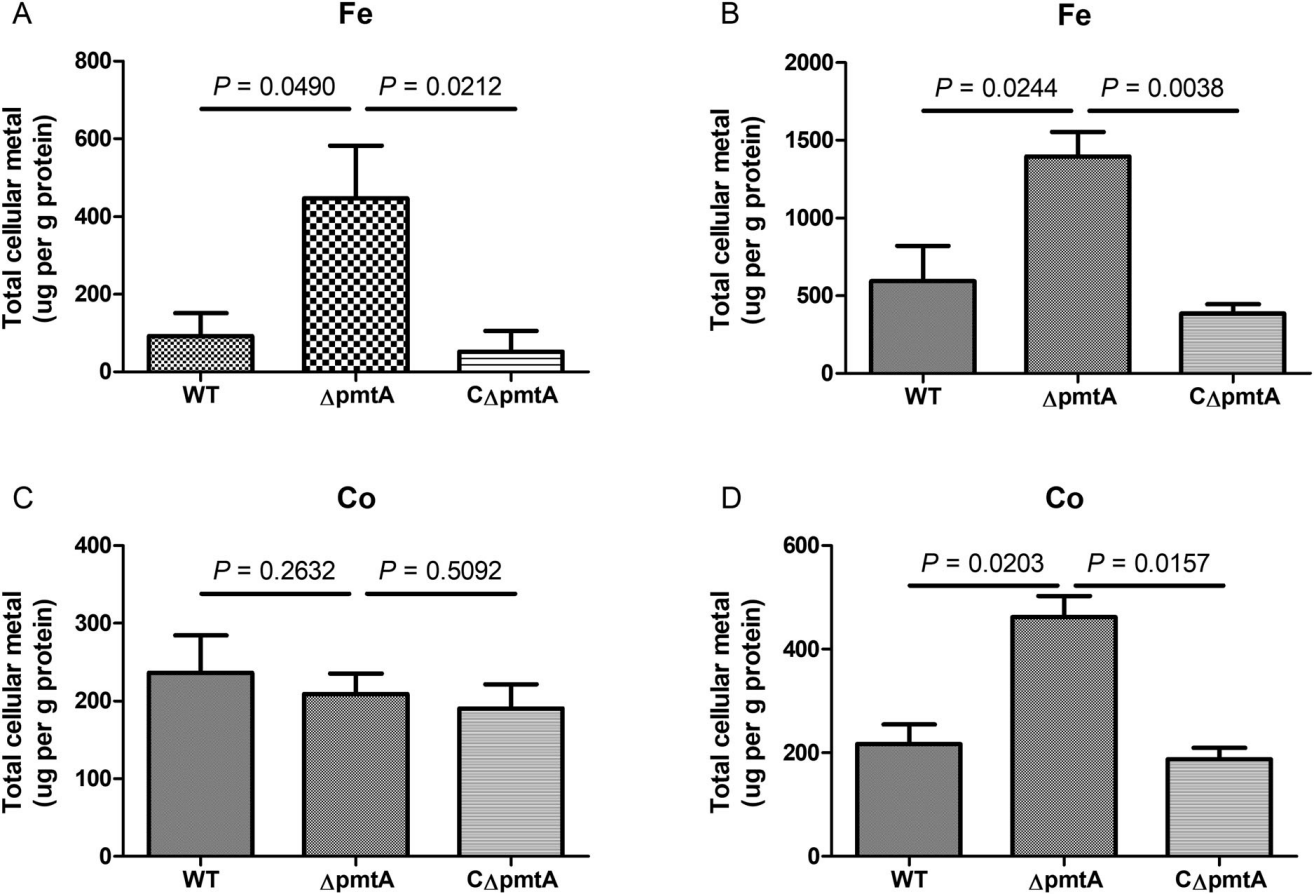
Levels of intracellular iron and cobalt in the WT, Δ*pmtA*, and CΔ*pmtA* strains. (A–B) Intracellular iron content in the absence (A) and presence (B) of Fe(II). (C–D) Intracellular cobalt content in the absence (C) and presence (D) of Co(II). Graphs data are mean values ± SD from three biological replicates.

To understand the basis of growth defect of Δ*pmtA* under Fe(II) or Co(II) excess conditions, we used ICP-OES to analyze the intracellular metal levels in the WT, Δ*pmtA*, and CΔ*pmtA* strains grown in the absence or presence of Fe(II) or Co(II). Both in the absence and presence of Fe(II), Δ*pmtA* accumulated significantly increased levels of intracellular iron (Figure 4A–B). In the absence of Co(II), the intracellular cobalt contents were similar in the three strains (Figure 4C). Nevertheless, following the addition of Co(II) to the growth medium, the level of intracellular cobalt accumulated was approximately 2-fold higher in Δ*pmtA* than in the WT and CΔ*pmtA* strains (Figure 4D). These results indicate that PmtA plays a role in the efflux of both iron and cobalt.

### The ΔpmtA mutant is more sensitive to the Fe(II)-activated antibiotic streptonigrin

**Figure 5.**
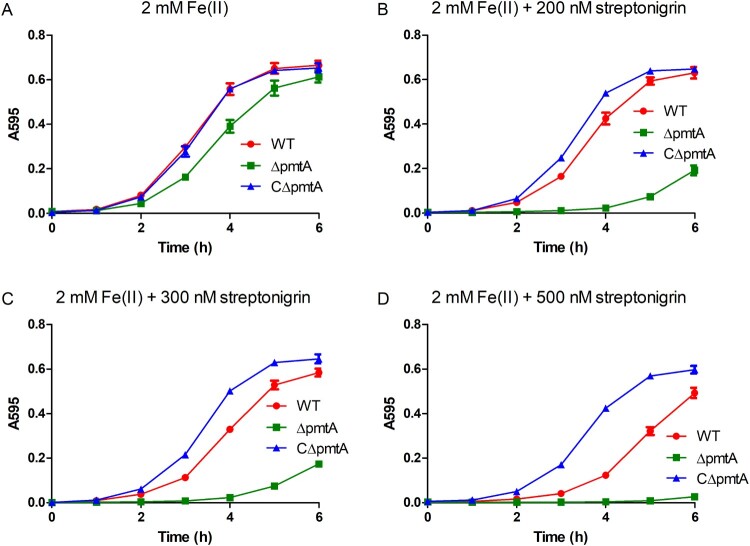
Δ*pmtA* exhibits increased sensitivity to streptonigrin. The *S. suis* strains were grown in the presence of 2 mM Fe(II) and increasing concentrations of streptonigrin. (A) No streptonigrin, (B) 200 nM streptonigrin, (C) 300 nM streptonigrin, and (D) 500 nM streptonigrin. Graphs data are mean values ± SD from three wells.

Streptonigrin is an antibacterial agent that requires iron for its bactericidal activity [36]. Since Δ*pmtA* accumulates increased levels of intracellular iron, we hypothesized that this mutant would be more sensitive to streptonigrin than the WT or CΔ*pmtA* strains. When grown in medium supplemented with 2 mM Fe(II), Δ*pmtA* showed only a slight growth defect (Figure 5A). In stark contrast, Δ*pmtA* exhibited a remarkable growth defect when grown in medium supplemented with 2 mM Fe(II) and 200 nM streptonigrin (Figure 5B). Moreover, the growth defect of Δ*pmtA* was even more severe when the concentration of streptonigrin was increased to 300 nM (Figure 5C), while growth was almost completely inhibited when the concentration was increased to 500 nM (Figure 5D). Interestingly, CΔ*pmtA* grew much better than the WT strain following the addition of streptonigrin to the medium (Figure 5B–D). These results suggest that *pmtA* deletion can sensitize *S. suis* to streptonigrin, while overexpression protects against the drug.

### Growth defect of ΔpmtA under Fe(II) or Co(II) excess conditions can be alleviated by Mn(II) supplementation

**Figure 6.**
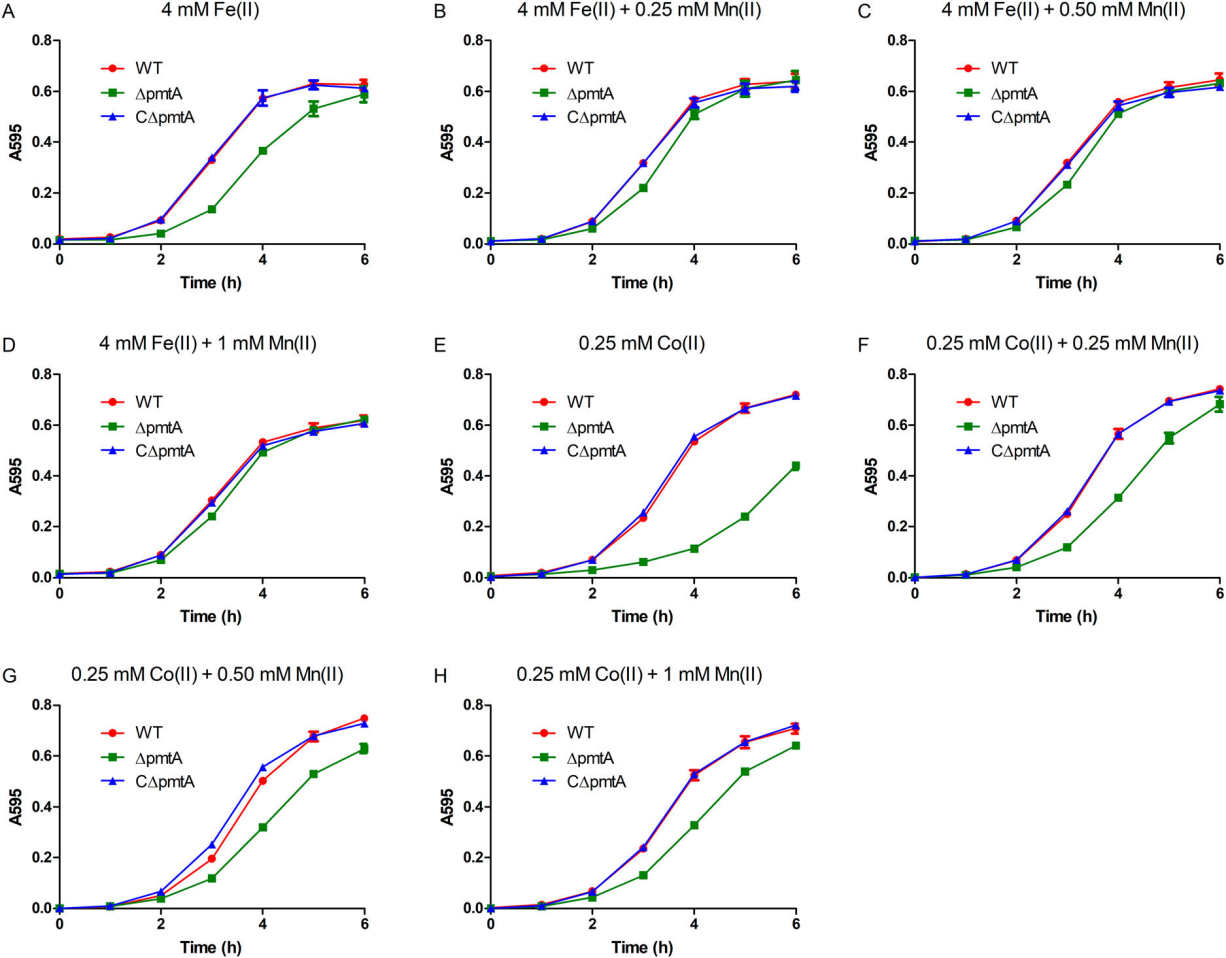
Mn(II) supplementation rescues growth defect of Δ*pmtA*. (A–D) The WT, Δ*pmtA*, and CΔ*pmtA* strains were grown in the presence of 4 mM Fe(II) alone (A) or 4 mM Fe(II) with 0.25 mM (B), 0.50 mM (C), or 1 mM Mn(II) (D). (E-H) Growth curves in the presence of 0.25 mM Co(II) alone (E) or 0.25 mM Co(II) with 0.25 mM (F), 0.50 mM (G), or 1 mM Mn(II) (H). Graphs data are mean values ± SD from three wells.

Previous studies have shown that in *B. subtilis* and *S. pyogenes*, inactivation of the Fe(II) efflux pump results in growth defects, which could be rescued by Mn(II) supplementation [7,11]. Based on these observations, we sought to determine whether the Fe(II) and Co(II) toxicity observed in Δ*pmtA* could be alleviated by Mn(II) supplementation. While the growth of Δ*pmtA* was inhibited by 4 mM Fe(II) (Figure 6A), the addition of Mn(II) to the medium rescued the growth of Δ*pmtA* under Fe(II) excess conditions (Figure 6B–D). Similarly, growth defect of Δ*pmtA* under Co(II) excess conditions was alleviated following Mn(II) supplementation (Figure 6E–H).

### PmtA plays a role in oxidative stress resistance in S. suis

**Figure 7.**
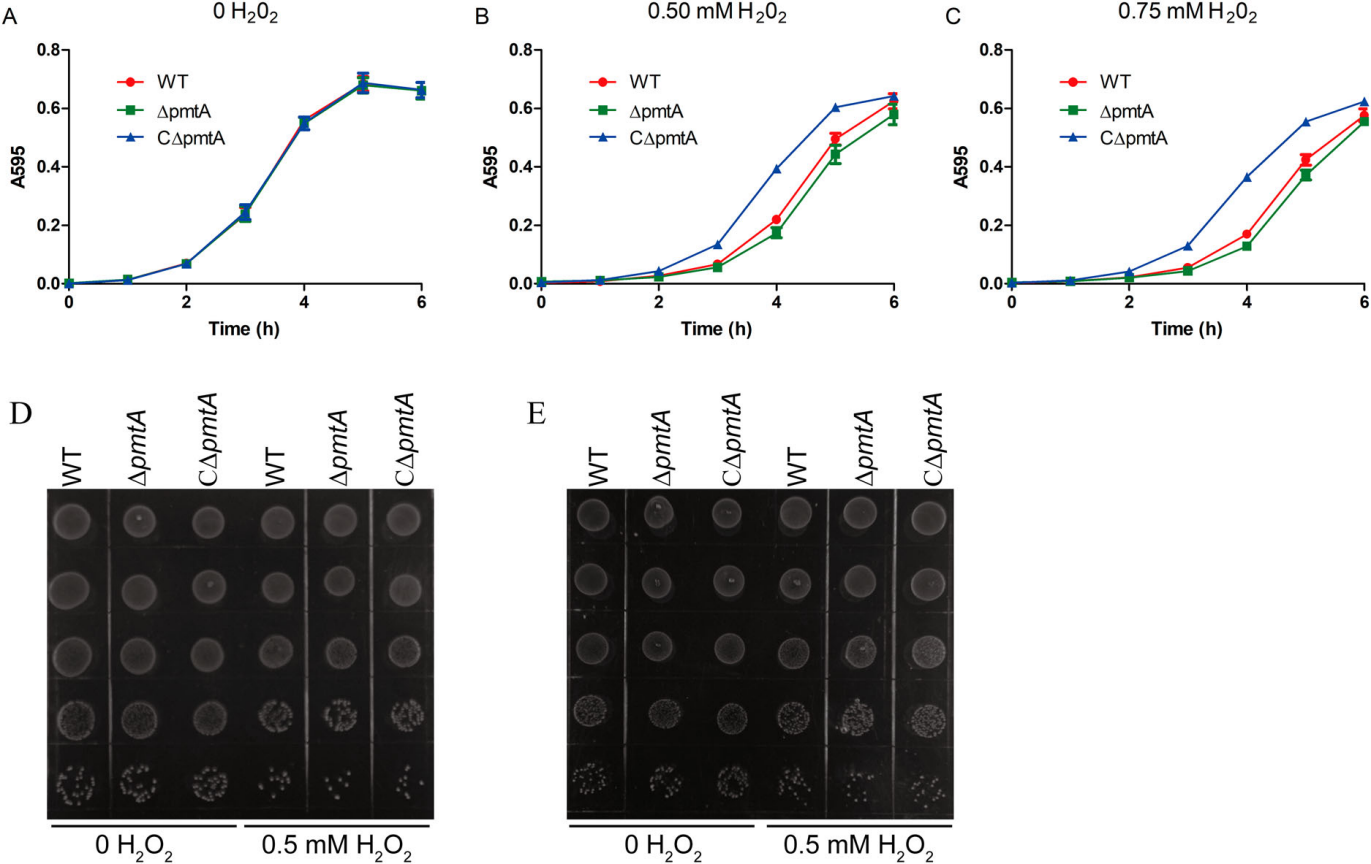
PmtA is involved in *S. suis* resistance to H_2_O_2_-induced oxidative stress. The *S. suis* strains were grown to exponential phase in the presence of 2 mM FeSO_4_. The cells were collected, diluted in fresh medium, and grown in the absence (A) and presence of 0.5 mM (B) or 0.75 mM H_2_O_2_ (C). Graphs data are mean values ± SD from three wells. (D–E) Spot dilution assays of the *S. suis* strains grown in the presence of 0.5 mM H_2_O_2_. At 4 h (D) and 6 h (E), aliquots were removed from the cultures, serially diluted 10-fold up to 10^–5^ dilution, and 5 μl of each dilution was spotted onto the plates from 10^–1^ (top) to 10^–5^ (bottom). The graphs are representative of three independent experiments.

Previous studies have revealed that the Fe(II) efflux mediated by PmtA is important for oxidative stress resistance in *S. pyogenes* [10,11]. We, therefore, examined the role of *S. suis* PmtA in oxidative stress tolerance using growth curve analyses. The WT, Δ*pmtA*, and CΔ*pmtA* strains were pretreated with 2 mM Fe(II) and then diluted in fresh medium supplemented with individual oxidative agents. The growth curves of all three strains were almost identical when grown in normal medium (Figure 7A). In medium supplemented with H_2_O_2_, Δ*pmtA* exhibited a moderate growth defect, while CΔ*pmtA* grew better than the WT strain (Figure 7B–C). Δ*pmtA* grown in the presence of 0.5 mM H_2_O_2_ also formed less colonies (Figure 7D–E). In contrast, the three strains exhibited similar growth in medium supplemented with diamide (Figure S7A) or paraquat (Figure S7B). We also examined the sensitivity of the WT, Δ*pmtA*, and CΔ*pmtA* strains towards these oxidative stress agents in the presence of 0.125 mM Co(II). We did not observe major differences when we compared the growth of these strains in medium supplemented with Co(II) alone and Co(II) with an oxidative agent (Figure S7C–F). Together, these results suggest that *S. suis* PmtA plays a role in tolerance to H_2_O_2_-induced oxidative stress.

### PmtA deletion does not affect S. suis virulence in mice

**Figure 8.**
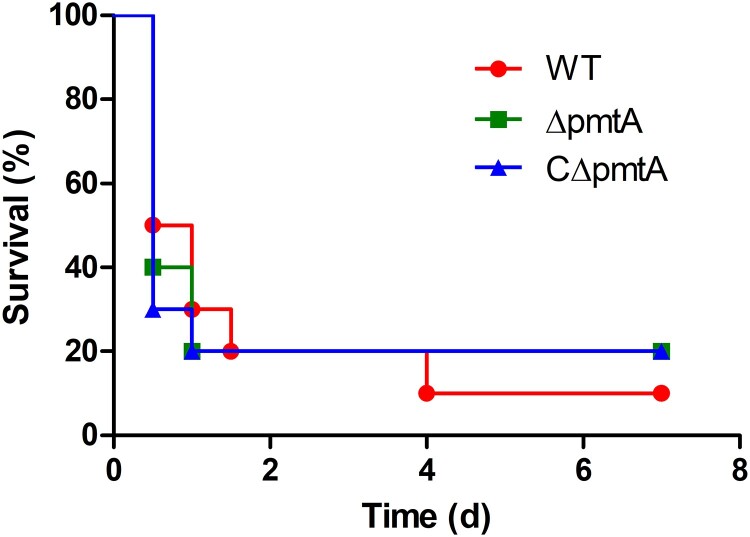
Survival curves of mice infected with the WT, Δ*pmtA*, and CΔ*pmtA* strains. Four groups of ten mice were inoculated intraperitoneally with PBS or 3×10^8^ CFU of the WT, Δ*pmtA*, or CΔ*pmtA* strains. No significant diﬀerence was observed between the different groups.

To evaluate the role of PmtA in *S. suis* virulence, we conducted an experimental infection study of BALB/c mice. Four groups of ten mice were inoculated intraperitoneally with PBS as a control or 3×10^8^ CFU of the WT, Δ*pmtA*, or CΔ*pmtA* strains. Following injection, the mice in the PBS group exhibited no sign of infection and survived through the experiment. However, the mice challenged with *S. suis* developed typical clinical symptoms, such as lethargy and prostration, within 12 h post-infection. Most of the infected mice died during the following days. The final survival rates for the WT, Δ*pmtA*, and CΔ*pmtA* groups were 10%, 20%, and 20%, respectively (Figure 8). We did not find significant differences between the survival rates of the Δ*pmtA* and WT groups (*P* = 0.9088), and those of the Δ*pmtA* and CΔ*pmtA* groups (*P* = 0.8690). Therefore, our data strongly suggest that PmtA does not contribute to the virulence of *S. suis* in mice.

## Discussion

The acquisition of transition metals is critical for both bacterial survival and pathogenesis. In *S. suis*, previous studies have demonstrated that several metal acquisition systems are required for full virulence [35,37,38]. In contrast to metal acquisition, metal efflux has attracted much less attention. Only recently, we showed that MntE acts as a Mn(II) export system that contributes to *S. suis* virulence [23]. To better understand the physiology and pathogenesis of *S. suis*, other metal efflux systems should be investigated.

Here, we report that PmtA functions as an Fe(II) and Co(II) efflux pump in *S. suis*. We provided evidence that: (i) *S. suis* PmtA exhibits a high level of identity with Fe(II) efflux systems from *S. pyogenes*, *L. monocytogenes*, and *B. subtilis*, which can also efflux Co(II) [7,8,10,11]; (ii) *pmtA* expression is significantly upregulated in response to Fe(II) or Co(II); (iii) Δ*pmtA* is highly sensitive to Fe(II) or Co(II) excess; (iv) Δ*pmtA* accumulates increased level of intracellular iron and cobalt; (v) *pmtA* deletion increases the sensitivity to streptonigrin, a Fe(II)-activated antibiotic. We also showed that growth defects of Δ*pmtA* under Fe(II) or Co(II) excess conditions could be alleviated by Mn(II) supplementation. Further more, we demonstrated that PmtA is implicated in tolerance to H_2_O_2_-induced oxidative stress. Although *pmtA* expression was not induced by Fe(III), both growth curves and spot dilution assays showed that Δ*pmtA* exhibited growth inhibition under Fe(III) excess conditions. A possible explanation is that the upper regulator of PmtA could not sense Fe(III). Interestingly, similar results have been reported for PfeT, a homolog of PmtA [7]. In *B. subtilis*, a *pfeT* null mutant displayed increased sensitivity to Fe(III) excess, whereas the ATPase activity of PfeT could not be activated by Fe(III) [7]. Under Zn(II) excess conditions, Δ*pmtA* showed a small growth defect, while CΔ*pmtA* grew better than the WT strain. The involvement of PmtA in Zn(II) transport has also been described in the Δ*perR* mutant of *S. pyogenes* [9]. Fur is involved in iron uptake and storage in many bacteria [39]. The expression of Fur regulon might be different in Δ*pmtA*, which is defective in iron efflux. In *S. suis*, Fur is required for Zn(II) resistence [34]. Thus, we speculate that growth defect of Δ*pmtA* under Zn(II) excess conditions might be due to the difference in expression of Fur regulon. Conversely, treatment of *S. suis* with Ni(II) resulted in a remarkable upregulation of *pmtA* expression, yet Δ*pmtA* exhibited no growth defect under Ni(II) excess conditions. Unlike the PmtA homologs in certain bacteria that are regulated by PerR or Fur [7–11], PmtA is not under PerR (Fur) control in *S. suis* [33,34]. The DNA-binding sequence of *S. suis* AdcR corresponds to the TTAACNRGTTAA motif [40]. In *S. suis*, a TTAACTTAGTTAA sequence is 33 bp in front of *pmtA*; thus, *pmtA* might belong to AdcR regulon. In *S. pneumoniae*, the AdcR regulon was highly induced by Ni(II) [41]. Therefore, we speculated that the induction of *pmtA* under Ni(II) excess conditions might be due to AdcR, or that the involvement of PmtA in tolerance to Ni(II) excess might be masked by the presence of other Ni(II) efflux mechanisms in *S. suis*.

The maintenance of the Fe/Mn ratio is important for bacterial physiology. In *S. pyogenes*, the deletion of either the Mn(II) or Fe(II) efflux pump resulted in growth inhibition under metal excess conditions, which could be restored by the addition of another metal [6,11]. Moreover, the Fe(II) sensitivity of a *B. subtilis pfeT* null mutant can be suppressed by Mn(II) supplementation [7]. In line with these observations, our results showed that growth defect of Δ*pmtA* under Fe(II) excess conditions was partly rescued following Mn(II) supplementation. Similarly, the addition of Mn(II) to the growth medium was able to relieve Co(II) toxicity. A possible mechanism for Fe(II) and Co(II) toxicity is a mismetallation affecting the activity of enzymes that require other metal cofactors, such as Mn(II) [7,42]. This hypothesis is in line with our observation that the addition of Mn(II) to the growth medium was able to alleviate the toxic effects of Fe(II) and Co(II) excess.

Fe(II) toxicity is also closely related to the oxidative stress resulting from the Fenton reaction [43]. The reaction between Fe(II) and H_2_O_2_ generates highly reactive hydroxyl radicals, which can cause DNA damage and membrane stress [1]. It is, therefore, reasonable to speculate that PmtA contributes to *S. suis* resistance to oxidative stress by mediating the efflux of Fe(II). In support of this hypothesis, our results showed that following pretreatment with Fe(II), Δ*pmtA* grew less efficiently in the presence of H_2_O_2_. Similar observations have been made for the Fe(II) efflux pumps of *Salmonella enterica* [44] and *S. pyogenes* [10,11]. Also, it is not surprising that CΔ*pmtA*, in which *pmtA* expression is significantly increased, grew better than the WT strain under H_2_O_2_ stress conditions.

It is well established that certain metal efflux systems contribute to bacterial virulence [5,6,8,23]. However, the role of PmtA in streptococcal pathogenesis remains controversial [10,11]. While VanderWal et al. demonstrated that PmtA is critical for *S. pyogenes* virulence in two mouse models of invasive infection [10], Turner et al. reported that Δ*pmtA* exhibits no virulence defect in a mouse model of invasive disease [11]. Here, we examined the involvement of PmtA in *S. suis* virulence using a mouse model of infection. Our results clearly showed that PmtA does not affect the virulence of *S. suis* in mice. Consistent with this result, Arenas et al. recently demonstrated that *pmtA* (SSU0288) is downregulated at all sites of infection and plays no major role in *S. suis* survival within the host using the piglet infection model [45]. Based on these observations, it is conceivable that the host may not employ iron toxicity to control bacterial infections.

In conclusion, the results presented here clearly demonstrate that PmtA is a Fe(II) and Co(II) efflux pump in *S. suis*. We also show that Fe(II) efflux mediated by PmtA is involved in the resistance to oxidative stress, while PmtA has no effect on *S. suis* virulence in mice.

## Supplementary Material

Supplemental Material
