# Supplementary material for: PmtA functions as a ferrous iron and cobalt efflux pump in Streptococcus suis
Source: Emerg Microbes Infect. 2019 Aug 30;8(1):1254–64. doi: 10.1080/22221751.2019.1660233 (PMC7012047; doi:10.1080/22221751.2019.1660233)
Supplement: Supplemental Material [file TEMI_A_1660233_SM7043.zip › Table S1_final.docx]

**Table S1.** Bacterial strains and plasmids used in this study.

| Strain or plasmid | Relevant characteristics^a^ | Source or reference |
| --- | --- | --- |
| Strains |  |  |
| SC19 | Virulent *S. suis* strain isolated from the brain of a dead pig | 26 |
| Δ*pmtA* | *pmtA* deletion mutant of strain SC19 | This study |
| CΔ*pmtA* | Complemented strain *of* Δ*pmtA*; Spc^R^ | This study |
| DH5α | Cloning host for recombinant vector | TransGen |
| Plasmids |  |  |
| pSET4s | Thermosensitive suicide vector; Spc^R^ | 29 |
| pSET4s-Δ*pmtA* | Knockout vector for *pmtA* deletion | This study |
| pSET2 | *E. coli*-*S. suis* shuttle vector; Spc^R^ | 30 |
| pSET2-*pmtA* | pSET2 containing *pmtA* and its promoter | This study |

^a^ Spc^R^, spectinomycin resistant.
