# Supplementary material for: PmtA functions as a ferrous iron and cobalt efflux pump in Streptococcus suis
Source: Emerg Microbes Infect. 2019 Aug 30;8(1):1254–64. doi: 10.1080/22221751.2019.1660233 (PMC7012047; doi:10.1080/22221751.2019.1660233)
Supplement: Supplemental Material [file TEMI_A_1660233_SM7043.zip › Table S2_final.docx]

**Table S2.** Primers used in this study.

| Primer | Sequence (5'-3')^a^ | Size (bp) | Target gene |
| --- | --- | --- | --- |
| QpmtA1 | CGATGTTGTCCGCAATGTCAC | 118 | an internal region of *pmtA* |
| QpmtA2 | AACCATCGCTTCTCCTTGTGC |  |  |
| Q16S1 | TAGTCCACGCCGTAAACGATG | 159 | an internal region of 16S rRNA |
| Q16S2 | TAAACCACATGCTCCACCGC |  |  |
| U1 | ACGC**GTCGAC**ACTCGCTGTTCTCGGTTTC | 740 | The upstream region of *pmtA* |
| U2 | ACCGCTCTCTATCAACCTCCCACTCGG |  |  |
| D1 | GGTTGATAGAGAGCGGTCGTGATGATGT | 627 | The downstream region of *pmtA* |
| D2 | CG**GAATTC**TGAACACGTCATCCAACATCT |  |  |
| In1 | GTCTTTTCTTGGTCACCCG | 755 | an internal region of *pmtA* |
| In2 | TCAAAAACAGCGACTTTCATT |  |  |
| Out1 | AGAAATAGCGAACACAAGCAG | 2327/600 | a fragment containing *pmtA* |
| Out2 | TAGAAATCCCGAGCTTGAAG |  |  |
| C1 | AAAA**CTGCAG**GACGAATATCAAAGCGGCTTTC | 2029 | *pmtA* and its promoter |
| C2 | CG**GGATCC**CTATTTAAAATACAATAGTCTCAACCCATT |  |  |

^a^ The bold sequences are restriction sites.
