# Supplementary material for: PmtA functions as a ferrous iron and cobalt efflux pump in Streptococcus suis
Source: Emerg Microbes Infect. 2019 Aug 30;8(1):1254–64. doi: 10.1080/22221751.2019.1660233 (PMC7012047; doi:10.1080/22221751.2019.1660233)
Supplement: Supplemental Material [file TEMI_A_1660233_SM7043.zip › Table S3_final.docx]

**Table S3.** Sequence identity of the *pmtA* gene in *S. suis*.

| *S. suis* strains | Locus tag | Gene sequence identity (%)^a^ |
| --- | --- | --- |
| CS100322 | CR541_01600 | 100 |
| LSM102 | A9494_01575 | 100 |
| SC19 | B9H01_RS01605 | 100 |
| SS2-1 | BVD85_01460 | 100 |
| ZY05719 | ZY05719_01555 | 100 |
| SC070731 | NJAUSS_0296 | 100 |
| JS14 | SSUJS14_0294 | 100 |
| GZ1 | SSGZ1_0284 | 100 |
| P1/7 | SSU0288 | 100 |
| SC84 | SSUSC84_0277 | 100 |
| 98HAH33 | SSU98_0305 | 100 |
| S735 | YYK_01355 | 99 |
| BM407 | SSUBM407_0279 | 99 |
| A7 | SSUA7_0289 | 99 |
| 05ZYH33 | SSU05_0309 | 99 |
| 6407 | ID09_01585 | 97 |
| T15 | T15_0314 | 96 |
| SS12 | SSU12_0292 | 96 |
| GZ0565 | BFP66_01540 | 96 |
| DN13 | A6M16_01655 | 96 |
| HN105 | DF184_01670 | 96 |
| SH0104 | - | 96 |
| HA0609 | CR542_01770 | 96 |
| 90-1330 | AN924_07850 | 96 |
| NSUI060 | APQ97_09520 | 96 |
| NSUI002 | AA105_01760 | 96 |
| 05HAS68 | HAS68_0291 | 96 |
| D9 | SSUD9_0340 | 96 |
| ST3 | SSUST3_0321 | 96 |
| TL13 | TL13_0343 | 96 |
| ST1 | SSUST1_0313 | 96 |
| YB51 | YB51_1565 | 96 |
| ISU2812 | A7J09_02680 | 95 |
| SH1510 | DP111_01660 | 95 |
| 1081 | BKM67_01660 | 95 |
| 0061 | BKM66_01655 | 95 |
| CZ130302 | CVO91_02020 | 94 |
| HN136 | CWM22_01935 | 94 |
| SRD478 | A7J08_01825 | 92 |
| HA1003 | DP112_01615 | 92 |
| D12 | SSUD12_0293 | 91 |
| AH681 | CWI26_01820 | 92 |

^a^ Gene sequence identity is compared with the *pmtA* gene of SC19 strain.
