# Supplementary figures and images for: PmtA functions as a ferrous iron and cobalt efflux pump in Streptococcus suis
Source: Emerg Microbes Infect. 2019 Aug 30;8(1):1254–64. doi: 10.1080/22221751.2019.1660233 (PMC7012047; doi:10.1080/22221751.2019.1660233)

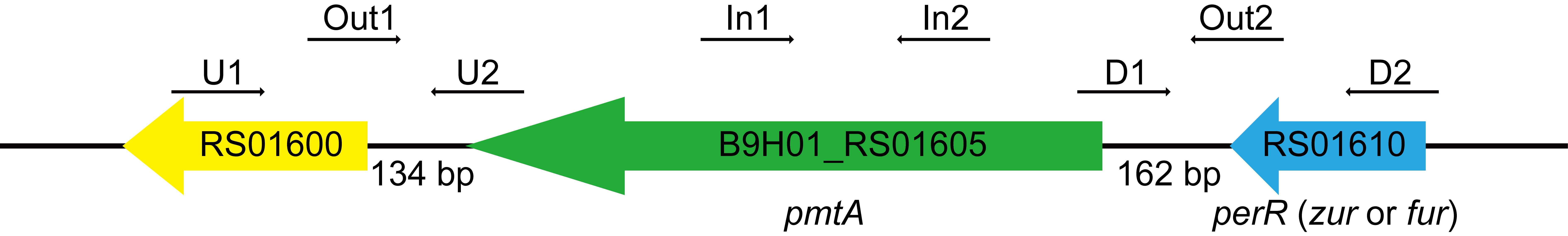

Supplement: Supplemental Material [file TEMI_A_1660233_SM7043.zip › Figure S2_final.tif]

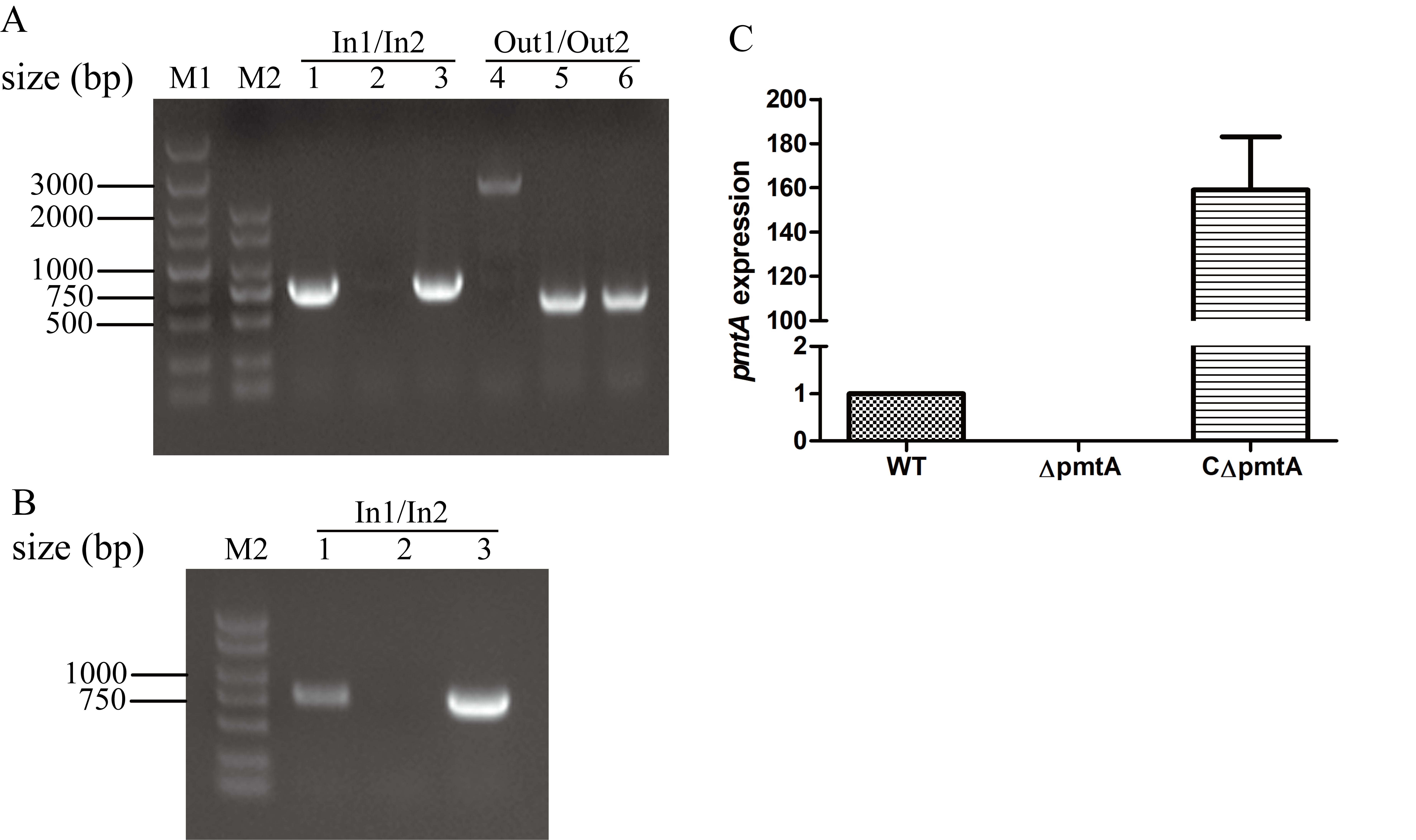

Supplement: Supplemental Material [file TEMI_A_1660233_SM7043.zip › Figure S3_final.tif]

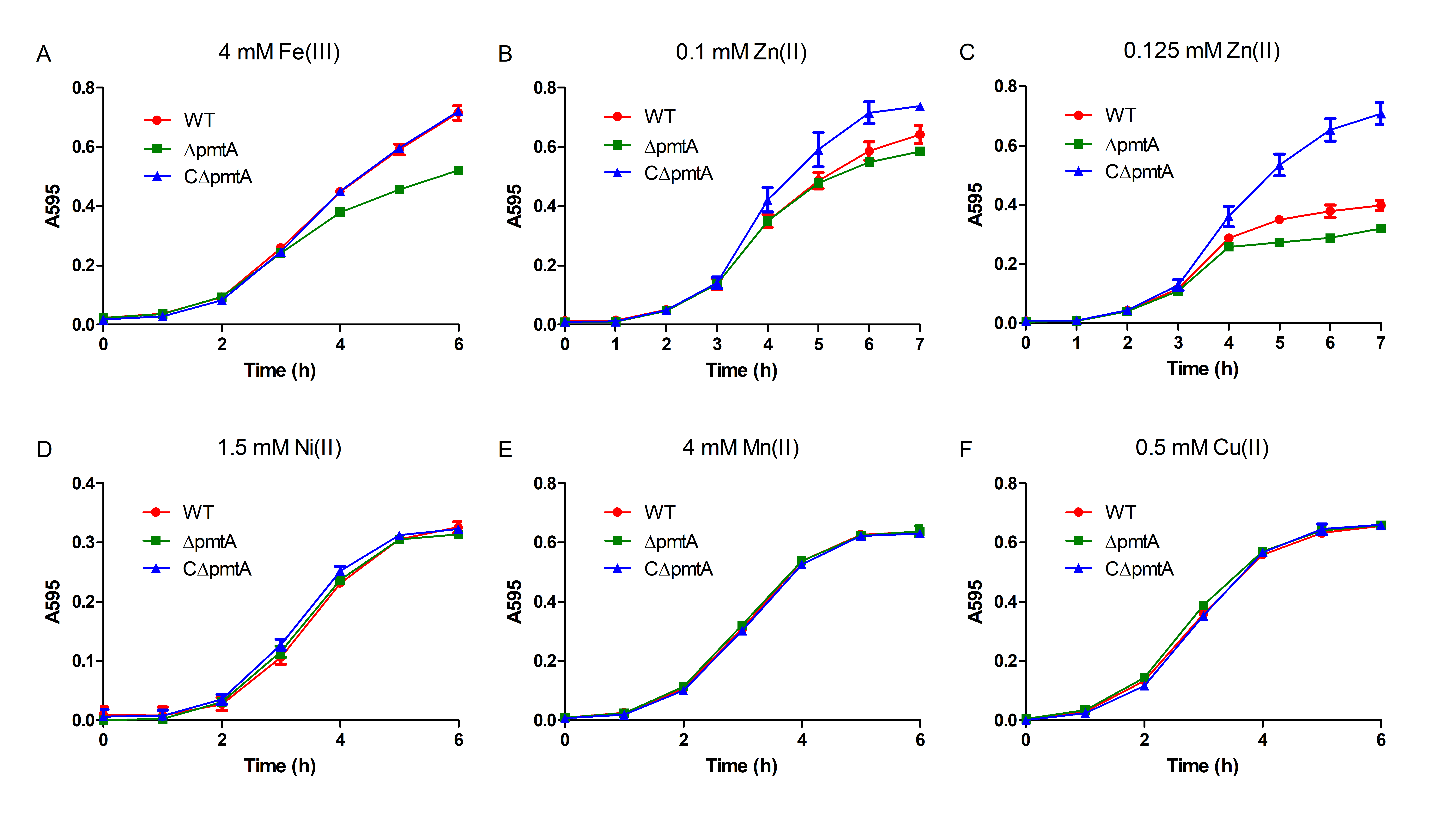

Supplement: Supplemental Material [file TEMI_A_1660233_SM7043.zip › Figure S4_final.TIF]

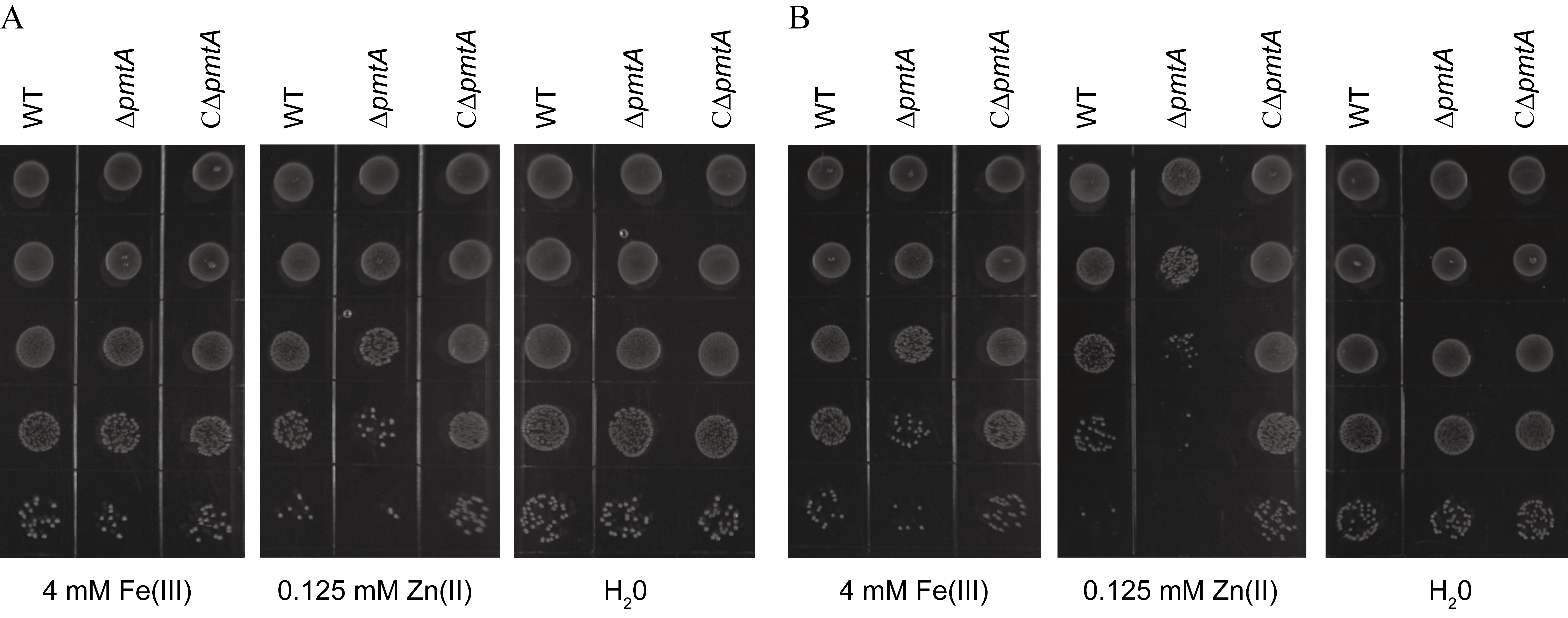

Supplement: Supplemental Material [file TEMI_A_1660233_SM7043.zip › Figure S5_final.tif]

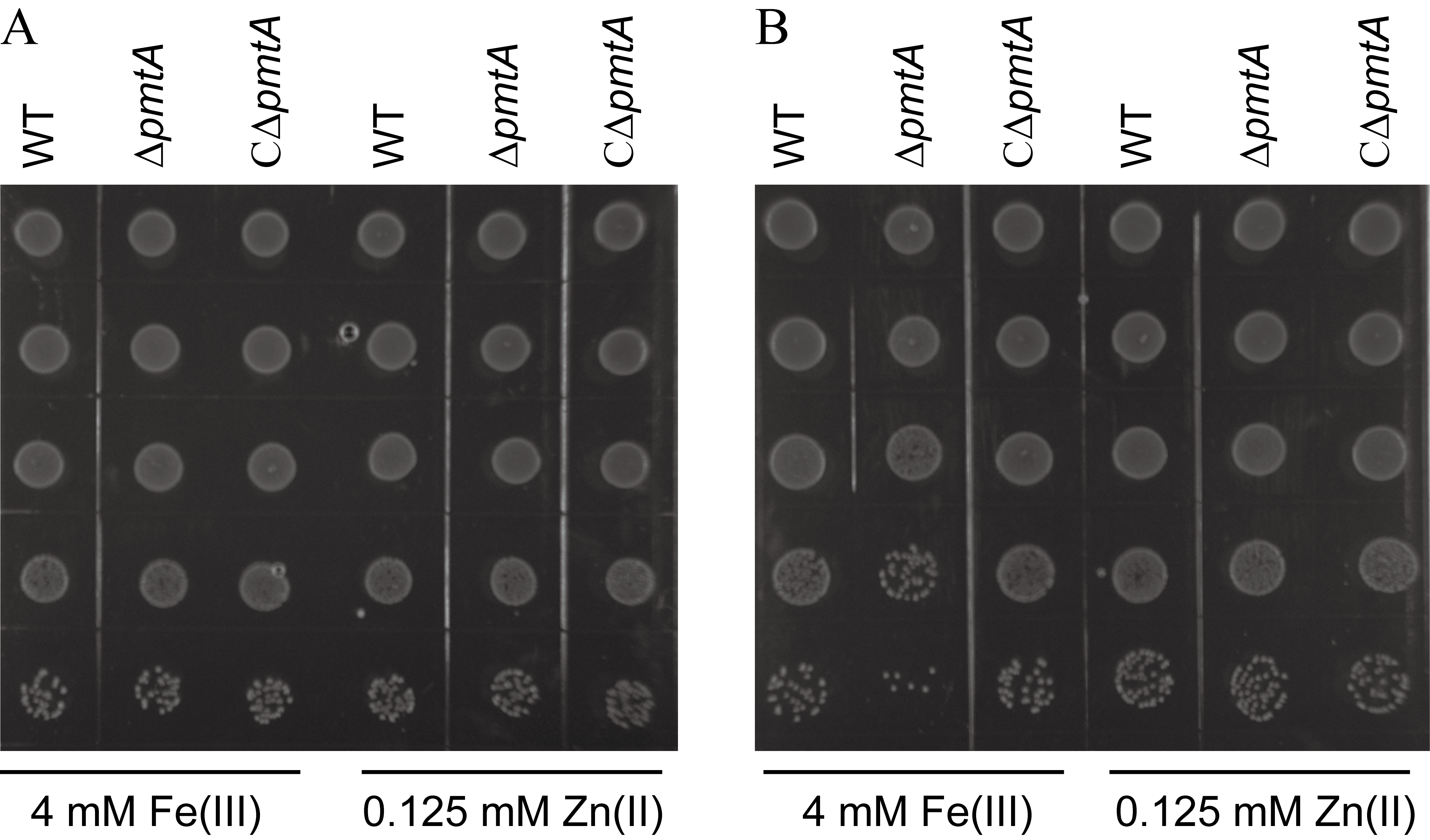

Supplement: Supplemental Material [file TEMI_A_1660233_SM7043.zip › Figure S6_final.tif]

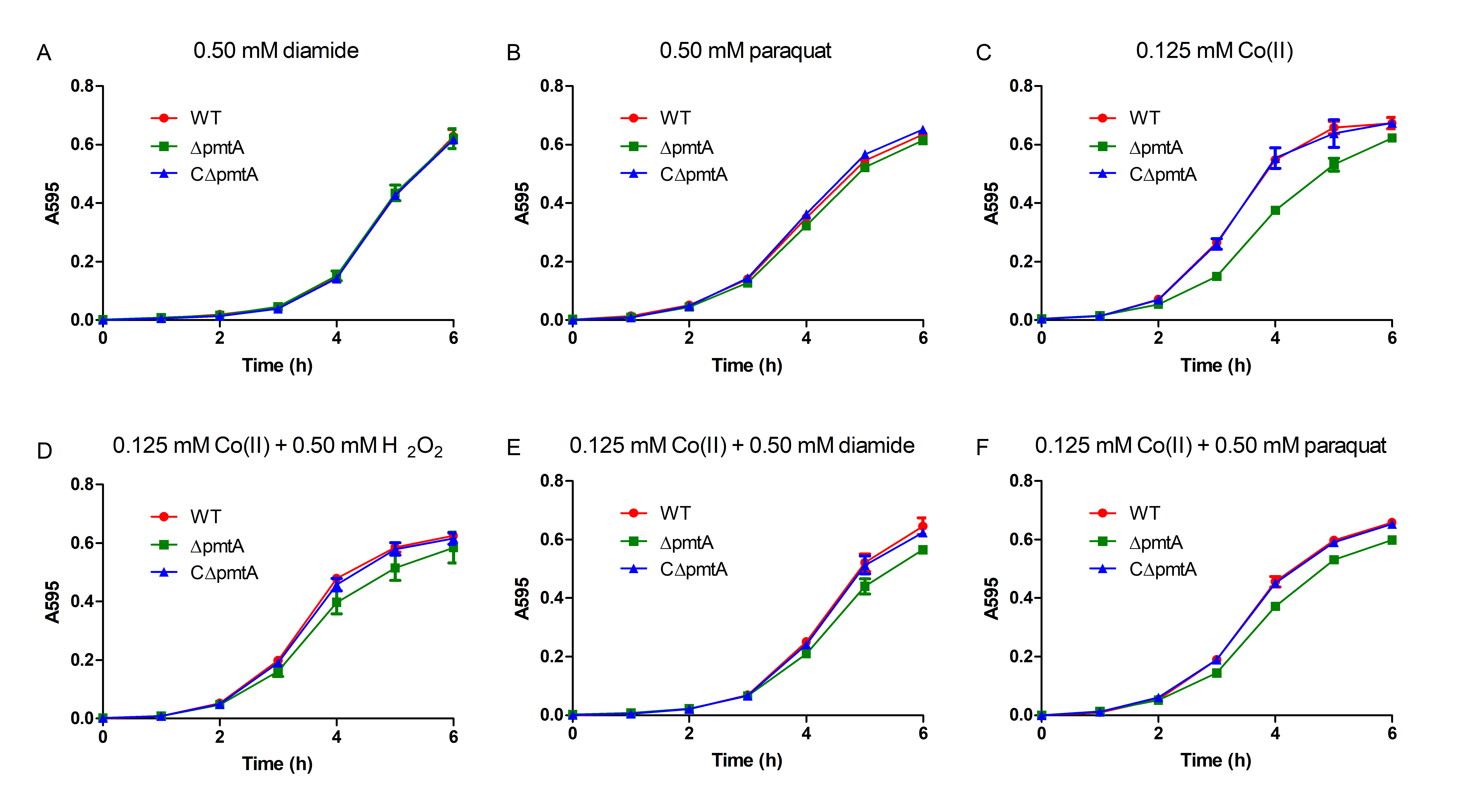

Supplement: Supplemental Material [file TEMI_A_1660233_SM7043.zip › Figure S7_final.TIF]
